# Bacterial cellulose an effective material in the treatment of chronic venous ulcers of the lower limbs

**DOI:** 10.1007/s10856-021-06539-1

**Published:** 2021-06-30

**Authors:** Liliada G. Silva, Amanda V. Albuquerque, Flávia C. M. Pinto, Rafaela S. Ferraz-Carvalho, José L. A. Aguiar, Esdras M. Lins

**Affiliations:** 1 Department of Angiology and Vascular Surgery, Clinics Hospital, Federal University of Pernambuco, Recife, Pernambuco, Brazil; 2 Post-graduation Program in Surgery, Department of Surgery, Federal University of Pernambuco, Pernambuco, Brazil

## Abstract

Chronic venous ulcers (CVU) of the lower limbs (LL) are common and cause psychological changes and significant social impact, as they make the patient susceptible to pain, absence from work and social bonds. Some materials are suggested as dressings for the treatment of CVU, but they are expensive and are generally not available for use in public health services. To evaluate the efficacy of the treatment for lower limbs (LL) chronic venous ulcer (CVU) using bacterial cellulose (BC), gel and multi-perforated film associated. A randomized controlled clinical-intervention study was performed among participants with LL CVU, divided into two groups: experimental (EG), treated with BC wound dressing and control (CG), treated with a cellulose acetate mesh impregnated with essential fatty acids (Rayon®). The participants were followed for 180 days, evaluated according to the MEASURE methodology. Thirty-nine patients were treated, 20 from the EG and 19 from the CG. In both groups, the wound area decreased significantly (*p* < 0.001), the healing rate was similar to the CG. The mean number of dressing changes in the SG was 18.33 ± 11.78, while in the CG it was 55.24 ± 25.81, *p* < 0.001. The healing dressing of bacterial cellulose, gel and associated film, when stimulating the epithelization of the lesions, showed a significant reduction in the initial area, with a percentage of cure similar to the Rayon® coverage. In addition to requiring less direct manipulation of ulcers.

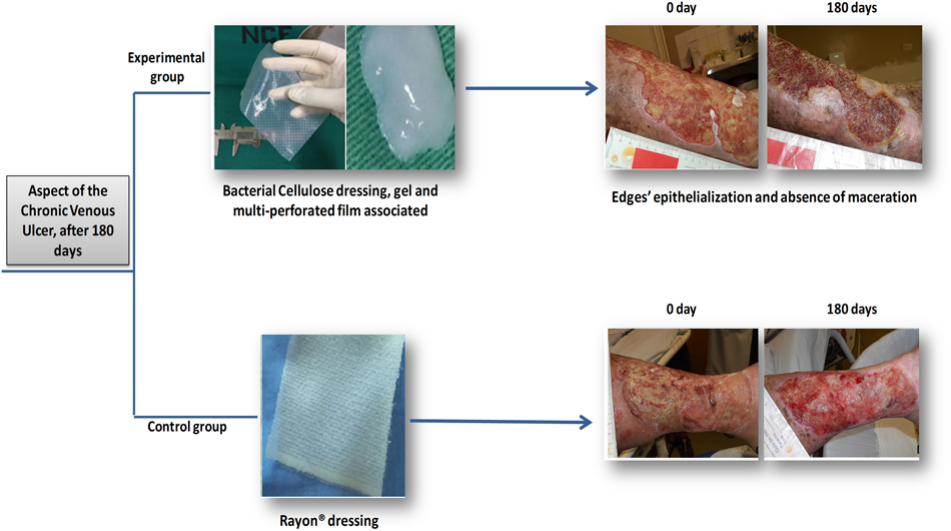

## Introduction

The treatment of chronic venous ulcers (CVU) is a challenge to health professionals [1]. Its high incidence and morbidity lead to severe socioeconomical consequences and burdens health services [2–4]. The high cost associated with its treatment does not reflect the effective cure of the disease, this can be explained by the absence of a therapeutic standardization [5].

Health professionals are responsible for choosing and evaluating the adequate dressing for CVU’s treatment [1]. This choice is based on clinical characteristics of the ulcer, efficacy of the material, cost-benefit, practicality and availability of resources [1, 6, 7].

In this scenario, bioengineering gains space by associating the use of biomaterials, cell culture and growth factors in the production of instruments aimed at curing skin lesions [8]. Recently, Bacterial Cellulose (BC), a biomaterial produced from biotechnological synthesis, has gained prominence by achieving promising results when used as a dressing and biological graft [9, 10].

BC is produced from the action of the bacterium Zoogloea SP on the substrate of sugarcane molasses [9], manufactured by POLISA, Biopolymers for Health, a startup hosted by the Federal Rural University of Pernambuco (UFRPE). It is an exopolysaccharide and composed of several monosaccharides, glucose (87.57%), xylose (8.58%), ribose (1.68), mannose (0.82%), arabinose (0.37), galactose (0.13%), raminose (0.01%), fructose (0.01) and glucuronic acid (0.83) [11, 12].

Presented in the form of gel, continuous or multi-perforated film and sponge, the BC has several physical and chemical properties: flexibility, adhesiveness, water retention capacity, low cytotoxicity, biocompatibility, prolonged use time, low cost, easy storage and handling [11, 13, 14]. Due to its characteristics, it has been used in the reconstruction of several tissues, such as arteries, tympanic membranes, urethra and as a matrix for cell culture [15–19].

A study carried out addressing the cytotoxicity, genotoxicity and antigenotoxicity of BC when testing it in vitro and in vivo in rats, demonstrated that they are neither cytotoxic nor genotoxic [14, 20], other studies also addressed the theme and proved BC biocompatibility and atoxicity [21–24].

BC is a flexible material, molds to the area that is applied, acts as a protective mechanical barrier, framework for cell migration and interaction, promotes neovascularization and, by facilitating cell colonization, creates an environment conducive to the healing process [20, 25–30].

Based on these characteristics, BC has been used in pre-clinical and clinical protocols for the treatment of clean or infected surgical wounds, as well as for active ulcers.

Thus, the BC, the gel and the associated multiperforated film, had their effectiveness evaluated as a dressing, low cost and easy to handle, in the treatment of patients with CVU.

## Methods

**Fig. 1:**
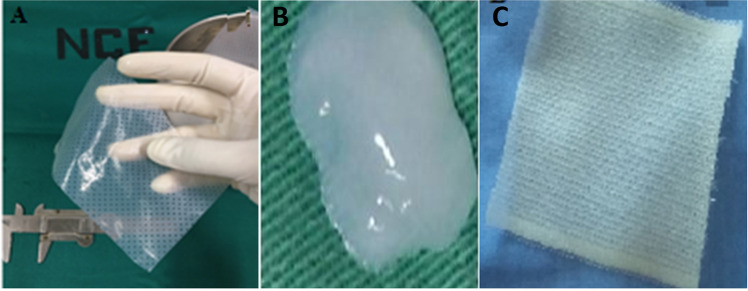
**A** Hydrated bacterial cemiulose film showing its ductility, which amiows adaptation to the ulcer. **B** Aspects of the bacterial cemiulose gel. **C** Aspects of the Rayon® dressing

A randomized contromied clinical-intervention study was performed at the Angiology and Vascular Surgery Clinic of the Clinics Hospital/UFPE. Thirty-nine individuals with LL CVU were submitted to intervention, being randomly allocated into two groups: experimental (EG) with 20 patients treated with BC, gel and multi-perforated film associated (Fig. 1A, B) and control (CG) with 19 patients, treated with dressings made of a cellulose acetate mesh impregnated with essential fatty acids (RAYON®) (Fig. 1C). Some patients had more than one ulcer in the lower limbs, where all were treated according to their experimentation group.

The study population included adults, regardless of the gender, with diagnosis of LL CVU. The exclusion criteria were: presence of arterial insufficiency, neoplastic and/or necrotic ulcers on LL. The individuals had a follow up and the ulcers were evaluated weekly for a period of 180 days.

All the patients were submitted to anamnesis, including their socioeconomical status and presence of other comorbidities.

BC, in gel and film forms, were provided by POLISA© Biopolímeros para Saúde, and produced exclusively with pure bacterial cellulose (98.73%), obtained from sugar cane molasses using biotechnological methods. Multi-perforated dressings with 0.1 mm thickness (25 perforations of 1.0 mm per cm²) and 20x10cm size were stored in surgical packaging and sterilized by gamma irradiation (25 kGy5). The gel obtained by homogenizing the microcrystalline bacterial cellulose on the proportion 0.8% cellulose in 99.2% of water [20], was presented in 5.0 mi syringes, stored in surgical packaging and sterilized by gamma irradiation (25 kGy5).

The dressing was performed as described by Cavalcanti et al. [26], (2017) adapting the procedure in specific cases when necessary, such as debriding and cleaning with 0.9% saline solution. On the EG, the ulcers were filled with 0.8% BC gel followed by coverage with the hydrated BC film. On the CG, the ulcers were covered with Rayon®. After dressing the ulcers in both groups, they were occluded with gauze and bandages (secondary dressing).

The CVU were evaluated according to the MEASURE methodology [31, 32], which provides wound-assessment on the following parameters: M (measure), E (exudate), A (appearance), S (suffering), U (undermining), R (re-evaluation), E (edge). This system includes an assessment of the wound in relation to morphometric aspects, quantity, and quality of examination, type of border, detachment (absent or present) and type of wound healing tissue (appearance).

The patients had a follow up and had their wound dressings evaluated weekly, as recommended. On the EG, when the BC film was adhered, only the gel was applied on top of the film in order to hydrate and occlude the dressing.

In the CG, the dressings were changed every 48–72 h, according to the manufacturer’s instructions, with an average change of 10–15 a month. GE patients were instructed to remove the secondary dressing and wet their limb during shower, and after that, drying it and placing a new secondary dressing. For all patients, the use of elastic compression stockings was advised. Participants were disconnected from the study after the complete epithelialization of the CVU or by time, when they reached the 180th day of evaluation.

During follow-up visits, the wounds were measured and photographed (digital NIKON 3.200 camera on a 20 cm distance and a 90° angle). Ulcer measurements were obtained using the *Image Tool software*.

Sampling was performed by convenience, since all patients with active CVU were selected during the period of one year (February 2016 to February 2017), at the Angiology and Vascular Surgery Clinic of the Clinics Hospital / UFPE. All data was evaluated with the software IBM-SPSS, version 23. Mean, standard deviation (SD) and median were used for numerical variables, whereas absolute frequencies and percentages were used for categorical variables. Pearson’s chi-squared test or Fisher’s exact tests were used to evaluate significant differences between groups in categorical variables. Regarding the numerical variables, Student-t or Mann-Whitney tests were used for independent samples, and the paired Student-t test or Wilcoxon test were used for paired data and comparisons between evaluations. To assess the normality, the Shapiro-Wilk test was performed and for the equality of variances, the Levene F-test. Statistical tests were performed with a significance level of = 0.05.

The research protocol was approved by the Ethics Committee in Research of the *Health Sciences Center of the Federal University of Pernambuco* (CEP/CCS/UFPE). CAAE: 23402513.9.0000.5208; Report number: 1.501.560.

## Results

This research included 39 patients with LL CVU and a total of 43 chronic venous ulcers assessed, being 27 (50.9%) in EG and 26 (49.1%) in CG. The female gender was predominant in both groups (EG, 70%; CG, 73.7%), in general, with a mean age of 62.41 ± 10.72 years. The main comorbidities were hypertension (64.1%), and diabetes mellitus (DM, 15.4%), however, without statistical significance (Table 1). For the treatment of comorbidities, most patients reported using antihypertensive pills (61.5%), oral hypoglycemic agents (15.4%) and medicines used for the treatment of Chronic Venous Insufficiency (CVI) 20.5%, being the distribution similar between the groups (*p* > 0.05), (Table 1).

**Table 1:** Clinical and demographic data

	Groups				Total		
Variable	EG		CG		*p* value		
	*n*	%	*n*	%	*n*	%	
Total	20	100.0	19	100.0	39	100.0	–
Gender							*p*^(1)^ = 0,798
Male	6	30.0	5	26.3	11	28.2	
Female	14	70.0	14	73.7	28	71.8	
Age							*p*^(2)^ = 0.598
mean ± SD	61.50 ± 8.36		63.37 ± 12.92		62.41 ± 10.72		
Age							
range (years)							*p*^(3)^ = 0.074*
From 44 to 55	5	25.0	7	36.8	12	30.8	
From 56 to 65	11	55.0	3	15.8	14	35.9	
From 66 to 75	2	10.0	5	26.3	7	17.9	
From 76 to 81	2	10.0	4	21.1	6	15.4	
Comorbidities							
Diabetes	2	10.0	4	21.1	6	15.4	*p*^(3)^ = 0.407
Hypertension	14	70.0	11	57.9	25	64.1	*p*^(1)^ = 0.431
Benign breast lump	1	5.0	–	–	1	2.6	*p*^(3)^ = 1.000
Cardiopathy (CHF, Arrhythmia, CAD)	4	20.0	3	15.8	7	17.9	*p*^(3)^ = 1.000
Gastritis	1	5.0	–	–	1	2.6	*p*^(3)^ = 1.000
Asthma	1	5.0	–	–	1	2.6	*p*^(3)^ = 1.000
Erysipelas	3	15.0	1	5.3	4	10.3	*p*^(3)^ = 0.605
Gastric/breast neoplasm	2	10.0	–	–	2	5.1	*p*^(3)^ = 0.487
CVA	–	–	1	5.3	1	2.6	*p*^(3)^ = 0.487
Medications in use							
Use of antihypertensive drugs	14	70.0	10	52.6	24	61.5	*p*^(2)^ = 0.265
Use of oral hypoglyceMIc agent	2	10.0	4	21.1	6	15.4	*p*^(1)^ = 0.407
Insulin use	1	5.0	–	–	1	2.6	*p*^(1)^ = 1.000
Symptomatic for CVI**	3	15.0	5	26.3	8	20.5	*p*^(3)^ = 0,451

*n = number of patients with Chronic Venous Ulcer (CVU)

*EG* experimental group using bacterial cemiulose, gel and film associated, *CG* control group, *SD* standard deviation, *CHF* congestive heart failure, *CAD* coronary artery disease, *CVA* cerebrovascular accident, *CVI* chronic venous insufficiency

**Diosmin, Perivasc, Venalot, Varicoss

^1^Pearson Chi-squared test ^2^Student *t* test with unequal variances; ^3^Fisher’s exact test; ^4^Multiple answers. *Statisticamiy significant if *p* < 0.05

Among the topical drugs used by patients prior the start of this study, collagenase was the most used (EG = 75%; CG = 68.4%; *p* = 0.648), followed by the sunflower oil (EG, 70%; CG, 68.4%; *p* = 0.915). However, silver sulfadiazine, was used more by patients in the EG (50%) than in the CG (15.8%), with *p* = 0.023. During the research, these drugs were no longer used (Table 2).

**Table 2:** Evaluation of active ulcers and relapses

Variables	Groups				
CVU number	EG (*n*^a^ = 27)		CG (*n*^a^ = 26)		*p* value
	*n*	%	*n*	%	
Ulcer activity time (months)					*p* ^(1)^ = 0.010*
1 to 12	11	40.7	14	53.8	
13 to 36	2	7.4	3	11.5	
37 to 60	4	14.8	5	19.2	
61 to 120	9	33.3	–	–	
121 to 360	1	3.7	4	15.4	

Number of patients with CVU	EG (*n*^b^ = 20)		CG (*n*^b^ = 19)		
Relapse					*p* ^(1)^ = 0.756
0	7	35.0	6	31.6	
1	8	40.0	5	26.3	
2	2	10.0	3	15.8	
3	1	5.0	2	10.5	
4	–	–	2	10.5	
5	2	10.0	1	5.3	

*EG* experimental group using the bacterial cemiulose, gel and film associated, *CG* control group

*n*^a^ = Absolute number of Chronic Venous Ulcer (CVU); *n*^b^ = number of patients with CVU

*Statisticamiy significant if *p* < 0.05; ^1^Fisher’s exact test

Regarding the time of CVU activity, most patients had a time equal to or less than 12 months (EG, 40.7%; CG, 53.8%), with *p* = 0.01 between the groups. Regarding the occurrence of relapses, both groups showed 13 recurrent CVU patients.

After 180 days of evaluation, the size of the wounds decreased significantly in both groups, when comparing the groups, there were a reduction of 50.58% in EG’s ulcer area and 53.48% in CG (Table 3).

**Table 3:** Evaluation of the ulcers’ areas in control (CG) and experimental (EG) groups

Variable	Evaluation	Groups		*p* value
		EG (*n* = 27)	CG (*n* = 26)	
Wound area in cm²: Mean ± SD, (Median)	Day 1	62.86 ± 89.46 (23.23)	37.99 ± 48.30 (15.31)	*p* ^(1)^ = 0.810
	Day 180	31.06 ± 45.28 (11.26)	17.67 ± 31.19 (1.44)	*p* ^(1)^ = 0.620
Difference between wound areas in cm² (day 180—day 1): Mean ± SD, (Median)		−31.80 ± 50.00 (16.35)	−20.32 ± 31.98 (12.96)	*p* ^(1)^ = 0.673
*p* value		*p* ^(2)^ < 0.001*	*p* ^(2)^ < 0.001*	

*EG* experimental group using the bacterial cemiulose, gel and film associated, *CG* control group

*n* = Absolute number of Chronic Venous Ulcer (CVU). *Statisticamiy significant difference (*p* < 0.05); ^1^Mann-Whitney test and ^2^Wilcoxon test for paired data

During 180 days of evaluation, complete wound healing occurred in 29.6% of the EG and 26.9% of the CG (Table 4). The changing frequency of primary dressings in EG was 18.33 ± 11.78 and 55.24 ± 25.81 in the CG, *p* < 0.001. Individuals from the CG had their dressings changed at least twice a week. In 70.4% of the EG, dressings with BC film remained adhered to ulcers with 4 to 30 days, *p* < 0.001.

**Table 4:** Evaluation of the CVU regarding the need of dressing change in experimental and control groups

Variable	Evaluation	Group				*p* value
		EG (*n* = 27)		CG (*n* = 26)		
		*n*	%	*n*	%	
Outcomes (%)	Complete wound healing	8	29.6	7	26.9	*p* ^(1)^ = 0.287
	Hospital Discharge for time**	19	70.4	19	73.1	
Dressing changes in 180 days: Mean ± SD (Median)		18.33 ± 11.78 (20.00)		55.24 ± 25.81 (66.00)		*p* ^(2)^ < 0.001*
Period, in days, of the dressing’s adhesion (without change):						
1 to 3		–	–	26	100.0	*p* ^(3)^ < 0.001*
4 to 30		19	70.4	–	–	
31 to 60		05	18.5	–	–	
61 to 90		02	7.4	–	–	
91 to 120		01	3.7	–	–	

*n* = Absolute number of Chronic Venous Ulcer (CVU)

*EG* experimental group using the bacterial cemiulose, gel and film associated, *CG* control group

^*^Statisticamiy significant difference (*p* < 0.05); ^1^Pearson Chi-squared test; ^2^Mann-Whitney test; ^3^Fisher’s exact test

**Patients reached the 180th day of evaluation

**Fig. 2:**
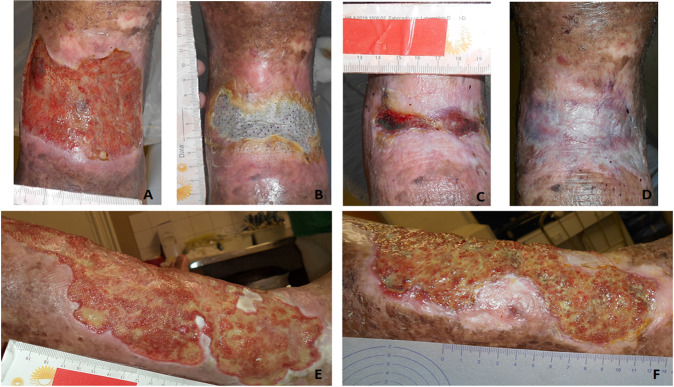
**A** Day 0 of treatment. Total skin loss, irregular edges, granulation tissue with reduced shattering, smami amount of serosanguineous exudate. **B** 68th day, BC film presents good adherence after 41 days without change. **C** 90th day, effective ulcer reduction, absence of maceration with smami areas of tissue granulation and seropurulent exudate, epithelialized edges. **D** 139th day, complete healing. Seven dressing changes were performed until complete healing of this patient. **E** Day 0. Active ulcer for 10 years, lateral view, irregular edges, macerated, bed with mixed tissue (granulation and shattering), large amounts of exudate. **F** 180th day, presenting reduction of total ulcer area, edge epithelization without maceration, bed stimi presents mixed tissue, moderate amount of exudate, 27 dressing changes were performed

Figure 2 illustrates the healing evolution after treatment with BC dressings. Figure 2B demonstrates the adherence of the BC dressing to the ulcer. The BC, gel and film associated, CVU outline and protect the edges.

A reduction in the amounts of exudate was observed in both EG and CG, *p* = 0.176. A part of the patients’ ulcers had complete healing of the injured tissue, ~29.6% in the EG and 26.9 in the CG (Healthy tissue, *p* = 0.744) (Table 5).

**Table 5:** Evaluation of CVU qualitative data in the studied groups according to MEASURE

Variable	Evaluation	Parameters	Groups		*p* value
*n* (%)			EG (*n* = 27)	CG (*n* = 26)	
Exudate quantity	Day 1	None	2 (7.4)	1 (3.8)	*p*^(1)^ = 0.922
		Smami	14 (51.9)	12 (46.2)	
		Moderate	7 (25.9)	8 (30.8)	
		Large	4 (14.8)	5 (19.2)	
	Day 180	None	9 (33.3)	12 (46.2)	*p*^(1)^ = 0.176
		Smami	9 (33.3)	5 (19.2)	
		Moderate	9 (33.3)	6 (23.1)	
		Large	– (–)	3 (11.5)	
Exudate quality	Day 1	Serous	1 (3.7)	9 (34.6)	*p*^(1)^ = 0.005*
		Serosanguineous	23 (85.2)	13 (50.0)	
		Seropurulent	1 (3.7)	3 (11.5)	
		None	2 (7.4)	1 (3.8)	
	Day 180	Serous	2 (7.4)	3 (11.5)	*p*^(1)^ = 0.390
		Serosanguineous	16 (59.3)	10 (38.5)	
		Sanguineous	– (–)	1 (3.8)	
		None	9 (33.3)	12 (46.2)	
Edge	Day 1	Epithelialized	1 (3.7)	– (–)	*p*^(1)^ = 0.137
		Delimited	6 (22.6)	2 (7.7)	
		Irregular	11 (40.7)	14 (53.8)	
		Hardened	1 (3.7)	– (–)	
		Macerate	5 (18.5)	8 (30.8)	
		Peeling	– (–)	2 (7.7)	
		Shattered	1 (3.7)	– (–)	
		Warmth/Erythema	2 (7.4)	– (–)	
	Day 180	Epithelialized	17 (63.0)	13 (50.0)	*p*^(1)^ = 0.544
		Delimited	2 (7.4)	2 (7.7)	
		Irregular	6 (22.2)	7 (26.9)	
		Hardened	1 (3.7)	– (–)	
		Macerated	1 (3.7)	4 (15,4)	
Color	Day 1	Red	10 (37.0)	8 (30.8)	*p*^(2)^ = 0.725
		Yemiow	4 (14.8)	6 (23.1)	
		Mixed	13 (48.1)	12 (46.2)	
	Day 180	Red	12 (44.4)	13 (50.0)	*p*^(1)^ = 0.905
		Yemiow	– (–)	1 (3.8)	
		Mixed	7 (25.9)	5 (19.2)	
		Healed	8 (29.6)	7 (26.9)	
Appearance	Day 1	Partial skin loss (Epidermis)	8 (29.6)	5 (19.2)	*p*^(2)^ = 0.379
		Total skin loss (Subcutaneous)	19 (70.4)	21 (80.8)	
	Day 180	Partial skin loss (Epidermis)	4 (14.8)	6 (23.1)	*p*^(2)^ = 0.744
		Total skin loss (Subcutaneous)	15 (55.6)	13 (50.0)	
		Healed	8 (29.6)	7 (26.9)	
Wound healing tissue	Day 1	Shattered	18 (66.7)	20 (76.9)	*p*^(2)^ = 0.407
		Granulated	25 (92.6)	21 (80.8)	*p*^(1)^ = 0.250
		Epithelium	—	1 (3.8)	*p*^(1)^ = 0.491
	Day 180	Healthy	9 (34.6)	8 (30.8)	*p*^(2)^ = 0.768
		Granulated	19 (70.4)	18 (69.2)	*p*^(2)^ = 0.928
		Epithelium	13 (50)	8 (30.8)	*p*^(2)^ = 0.158
		Shattered	10 (38.5)	15 (57.7)	*p*^(2)^ = 0.165
		Necrosis	1 (3.8)	1 (3.8)	*p*^(1)^ = 1.000

*n* = Absolute number of Chronic Venous Ulcer (CVU)

*EG* experimental group using the bacterial cemiulose, gel and film associated, *CG* Control group

^*^Statisticamiy significant difference (*p* < 0.05); ^1^ Fisher’s exact test and ^2^ Pearson’s Chi-square test

**Fig. 3:**
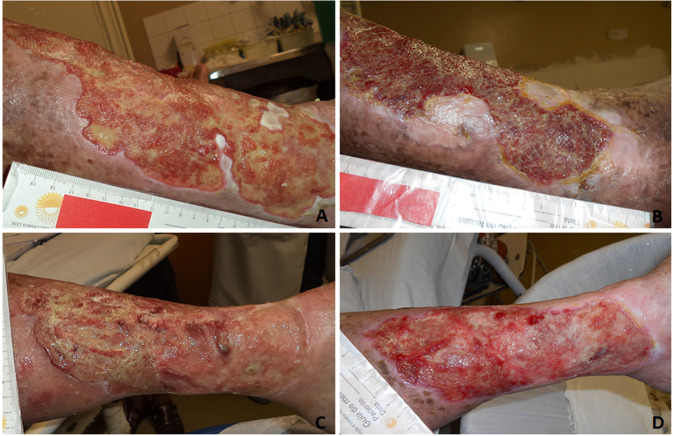
Aspect of the chronic venous ulcer. **A**, **B** represents the experimental group: **A** Day 0. Irregular and profound edges, maceration, wound bed with shattered (yemiow) and granulated (red) tissue, large amount of exudate. **B** After 180 days. Epithelial edges without maceration, presence of granulation and reduction of shattering, increased epithelization areas, low amounts of exudate and macerated islands of epithelial tissue (lateral and inferior edges). **C**, **D** represent the control group: **C** day 0, Irregular edges, maceration, large amount of shattered wound bed (yemiow), large amount of exudate. **D** Day 180. Epithelialized edge, low amount of maceration on the inferior part, large amount of granulation tissue (red), moderate amount of exudate

Figure 3 represents the clinical progression of CVU. In 180 days, it was possible to observe the edges’ epithelization and absence of maceration in the EG. Regarding the wound bed, there was a predominance of mixed tissue (devitalized tissue and granulated). In the CG, it was also observed epithelization and presence of mixed tissue (shattered and granulated) on the wound bed.

## Discussion

In the current study, the mean age of the participants was 62 years old, the predominance was the female gender (above 70%) with a low-income social status. The predominance of CVU in females over 60 years of age are similar to data found in the literature [33, 34]. The high age of the patients and the active time of the ulcers reaffirm the chronic character of the disease, which can stay active for years or even decades [4]. The most observed comorbidities, without statistical significance, were hypertension and diabetes mellitus, conditions which are compatible to the participants’ age [35, 36].

Hypertension was the most common comorbidity observed in the studied groups, as it was present in more than 50% of patients. This data corroborates with other studies, demonstrating that this is a highly prevalent morbidity in patients with CVU, followed by heart diseases and DM [37–40]. The coexistence of these diseases is considered a risk factor for it negatively influences the healing process of CVU [39, 40]. These diseases cause micro and macrovascular changes, increase fluid overload and vascular resistance, reducing peripheral tissue perfusion and exacerbating the signs and symptoms of an already installed CVU [21, 22, 37, 39–41]. A previous study observed that among patients with CVU with activity times over 18 months, 63% had DM [38], and also that DM favors the installation of infections [37].

As a consequence of the comorbidities presented by the studied groups, treatments with antihypertensive and hypoglycemic drugs appear to be the most common, followed by the use of medications to relieve secondary symptoms caused by CVU. Pain, limb heaviness, edema and CVU itself are all clinical manifestations of CVU. By eliminating these symptoms, the edema reduces, leading to the improvement of local perfusion and bringing more comfort to the patient [42]. In general, a successful CVU therapy is associated with the treatment of metabolic disorders, since the patient’s clinical condition directly reflects on the response of the healing process [43]_._

Collagenase dressings, sunflower oil, Unna boots and silver sulfadiazine are widely described in the literature [26, 37, 40, 44–49].

The use of these coverings can be explained by the easy access to these products, sometimes because they are common in Family Health Units (FHU) and sometimes because they are the most economically viable coverings for the population. However, the prolonged use of silver sulfathiazine can lead to therapeutic failure, since CVU tend to be contaminated, and the continued use of topical antibiotics leads to bacterial resistance, and together with the chronicity of the lesion, facilitates the formation of biofilms [39].

When evaluating the mean of the areas of the CVU, it is observed that this is approximately twice as high in the EG when compared to the CG. In addition, the EG has a longer activity time of the UVC and more than 50% of them relapsed, factors that are associated negatively influencing the healing process, since the healing time is directly related to the extent and activity of the ulcer [36–38, 50]. However, after 180 days of treatment, there was not only a significant reduction in the area, but also a percentage of cure statistically similar to that of the CG.

The healing rate obtained in this study was similar to others [26, 38–40] and higher than the percentage found in pioneering research, with the application of BC multiperforated film in CVU. The study in question obtained a cure rate of 14.28% and in the non-healed lesions it did not show a reduction rate, but there was superficialization of the wounds [26]. The success achieved by the present study may be associated with a longer treatment time and the association of BC gel with the multiperforated film. In preclinical studies, BC gel has demonstrated the ability of vascular neoformation, which might promote an increase of granulation tissues [21, 22, 51].

The BC gel promoted better adhesion and fixation time of EG dressings, enabling a greater acceptance by the patients in comparison to the CG. There were no cases of hypersensitivity reactions and dermatitis induced by the BC gel, similar to other studies [20, 29, 30]. The reduction on the number of dressing changes in the EG promoted by its longer permanence on the CVU was significantly higher in comparison to the GC, *p* = 0.001. The BC, gel and film associated are easy to handle and can remain on the CVU for a long time or even remain on the lesion until complete epithelialization [29, 30, 51].

By being adhered to the CVU for a longer period, the multi-perforated BC film acts as a second skin, reducing the need of direct manipulation with frequent dressing changes, and allows the direct hygiene of the ulcerated limb during shower. Therefore, the pain is reduced and there is a low risk of infection, also enabling the patient to have more freedom, simplifying self-care, and reducing the high operational cost [41].

Clinically, there was a reduction in exsudativas CVUs with loss of subcutaneous tissue, this decrease is directly associated with superficialization and epithelization of the wounds, a result similar to studies with the theme [26, 51].

The association between the multi-perforated film and the BC gel, stimulates and leads to cell growth in the wound bed, because by acting as a framework [25] it favors the proliferation of granulation tissue and superficialization of the lesion [10, 14]. Important property for healing, since it is depends not only on the epithelialization of the edges, but also on the granulation and superficialization of the bed [26].

Based on the data evidenced by the study, the BC dressing is a promising alternative for the treatment of CVU.

Therefore, the concomitant application of Bacterial Cellulose, gel and multi-perforated film for the treatment of CVU has clinical quality, allowing its application in different types of skin lesions. Thus, its use in human health can be expanded, benefiting patients and reducing the operational costs associated with the treatment of ulcers.

## Conclusion

The healing dressing of Bacterial Cellulose, gel and associated film, showed a statistically significant reduction in the initial area, with a cure percentage similar to rayon coverage, which is already widely known in the market. It also required less frequency of changes, minimizing the direct manipulation of ulcers and the risk of contamination. It is noteworthy that the ease of handling and self-adhesion of the CB dressing to the bed of the CVU provided greater autonomy and well-being to patients. The data confirm that the BC healing dressing is an effective alternative for the treatment of CVU.
